# A novel medium for long-term primary culture of hemocytes of *Metapenaeus ensis*

**DOI:** 10.1016/j.mex.2023.102335

**Published:** 2023-08-19

**Authors:** Yaqi Zhao, Huarong Guo

**Affiliations:** a Key Laboratory of Marine Genetics and Breeding, College of Marine Life Sciences, Ministry of Education, Ocean University of China, Qingdao 266003, China; b Institute of Evolution and Marine Biodiversity, Ocean University of China, Qingdao 266003, China

**Keywords:** Preparation of growth medium for shrimp cells, Shrimp, Hemolymph, Shrimp cell medium, Long-term cell culture

## Abstract

The development of a suitable shrimp cell medium is essential for achieving a long-term cell culture and finite cell line of shrimps routinely. In this study, we have successfully developed an optimal shrimp cell medium that can be used for long-term *in vitro* culture and continuous subculture of the hemolymph cells (or hemocytes) of greasyback shrimp *Metapenaeus ensis*, designated as MeH cells, by shrimp serum-based and supplements-based optimization of the basic and growth medium. In this article, we have focused on the details for the preparation of the optimal shrimp cell medium by diluting and mixing of various stock solutions as well as the methods for isolation and primary culture of MeH cells.

• A novel shrimp cell growth medium is developed for long-term shrimp hemocytes culture.
• The preparation method of shrimp cell growth medium is successfully established.
• Obvious cell activity and proliferation potential of isolated shrimp cells can be maintained beyond 30 days.

## Graphical abstract

**Figure:**
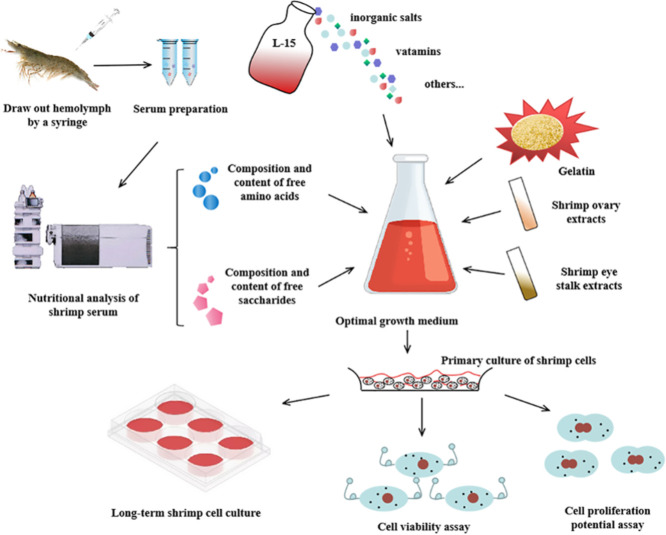

Specifications table

**Table:** 

Subject area:	Agricultural and Biological Sciences
More specific subject area:	Marine animal cell culture
Name of your method:	Preparation of growth medium for shrimp cells
Name and reference of original method:	N/A
Resource availability:	N/A

## Method details

The optimization of shrimp cell growth medium (SGM) was based on both the composition of l-15 medium and the nutrient composition and content of shrimp serum (free amino acids, free saccharides and free fatty acids, etc.). In detail, on the basis of l-15 medium components, the SGM medium was optimized by removing all the components of amino acids from l-15 medium and then sequentially adding varied folds of the shrimp serum-based amino acid mixtures, or shrimp serum-based saccharide mixtures, or shrimp serum-based fatty acid mixture and other organic and inorganic supplements such as FBS, gelatin, and so on, which listed in Table 1. A total of 16 kinds of shrimp cell basic media (SBM) and 16 kinds of SGMs had been prepared and tested to obtain the formulation of the optimal SGM. The detailed optimization process and evaluation of these media can be found in related paper titled by routine development of long-term primary cell culture and finite cell line from the hemolymph of greasyback shrimp (*Metapenaeus ensis*) and virus susceptibility [1]. In the present study only the detailed protocol for the preparation of the optimal SGM is addressed.

**Table 1:** The formulas of the optimal shrimp cell growth medium (SGM) [1].

Amino acids	mg L^−1^	Trehalose	229.02±4.77
γ-ABA	2.83±0.23	Gentiobiose	84.32±3.77
Serine	35.52±3.93	*D*+ Galactose	900
Valine	18.08±1.99		
Lysine	153.60±17.25	**Vitamins**	**mg L^−1^**
Cystine	21.65±1.55	i-Inositol	2
Leucine	81.05±9.15	Folic acid	1
Glycine	154.84±17.36	Niacinamide	1
Taurine	747.18±81.92	Choline chloride	1
Proline	1365.56±152.74	D-Calcium pantothenate	1
Alanine	184.85±20.60	Pyridoxine hydrochloride	1
Tyrosine	5.58±0.38	Thiamine monophosphate	1
Ornithine	16.56±1.84	Riboflavin 5′-phosphate Na	0.1
Arginine	255.58±28.57		
Histidine	26.38±1.58	**Inorganic salts**	**mg L^−1^**
Isoleucine	50.89±5.66	KCl	400
Threonine	13.05±1.46	NaCl	8000
β-Alanine	4.33±0.23	CaCl_2_	140
Methionine	7.03±0.77	MgCl_2_	93.7
Asparagine	147.01±16.49	MgSO_4_	97.7
Aspartic acid	48.51±5.39	KH_2_PO_4_	60
Glutamic acid	88.59±9.81	Na_2_HPO_4_	190
Phenylalanine	39.1 ± 4.35	NaHCO_3_	1000
Hydroxyproline	56.29±6.26		
Glutamine	300	**Other supplements**	**Per liter**
L-Cysteine	120	FBS	150 mL
L-Tryptophan	20	Gelatin	86 g
		Phenol Red	10 mg
**Saccharides**	**mg L^−1^**	Streptomycin	1.0 × 10^5^ IU
Xylose	4.68±0.84	amphotericin B	0.25 mg
Fucose	7.46±0.17	Sodium Pyruvate	550 mg
Ribose	25.67±1.85	Penicillin G sodium	1.0 × 10^5^ IU
Glucose	2920.37±15.18	Shrimp ovary extracts	200 mL
Sucrose	66.13±3.28	Shrimp eye stalk extracts	40 mL
Lactose	14.39±3.14	Epidermal growth factor	20 µg
Fructose	5.11±0.80	Basic fibroblast growth factor	20 µg
Arabinose	18.62±0.24		

*Note:* The medium listed in the table were adjusted to a osmolarity of 620 ± 20 mOsm∙kg^−1^ and pH value of 7.2 ± 0.2.

Importantly, the components of the optimal shrimp cell growth medium (SGM) included 26 kinds of amino acids, 12 kinds of saccharides, 8 kinds of vitamins, 8 kinds of inorganic salts, phenol red, 8.6% (m/v) gelatin, 20% (v/v) shrimp ovary extracts, 4% (v/v) shrimp eye stalk extracts, 15% (v/v) fetal bovine serum (FBS), 20 µg L^−1^ epidermal growth factor (EGF), 20 µg L^−1^ basic fibroblast growth factor (bFGF), 1.0 × 10^5^ IU L^−1^ penicillin G sodium, 1.0 × 10^5^ IU L^−1^ streptomycin and 0.25 mg L^−1^ amphotericin B. The formulas of shrimp cell growth medium are listed in details in Table 1. The formulas of 12 kinds of stock solutions (A-L) used to prepare the optimal SGM is listed in Table 2.

**Table 2:** The formulas of stock solutions used to prepare the optimal SGM.

Stock solution and its components	content	Stock solution and its components	content
**Amino Acids**		Ribose	2567±185
**Stock solution A (100×, in water)**	**mg L^−1^**	Sucrose	6613±328
Glycine	15,484±1736	Lactose	1439±314
L-Serine	3552±393	Fructose	511±80
L-Valine	1808±199	Arabinose	1862±24
L-Lysine	15,360±1725	Trehalose	22,902±477
L-Alanine	18,485±2060	Gentiobiose	8432±377
β-Alanine	433±23	*D*+ Galactose	90,000
Ornithine	1656±184	Sodium pyruvate	55,000
L-Arginine	25,558±2857		
L-Threonine	1305±146	**Stock solution G (100×, in water)**	**g *L*** **^−^** **^1^**
Hydroxyproline	5629±626	Glucose	292±1.5
4-Hydroxybutanoic acid (γ-ABA)	283±23		
L-Glutamine	30,000	**Vitamins**	
		**Stock solution H (100×, in water)**	**mg *L*** **^−^** **^1^**
**Stock solution B (100×, in water)**	**g *L*** **^−^** **^1^**	i-Inositol	200
Taurine	74.7 ± 8.2	Niacinamide	100
		Choline chloride	100
**Stock solution C (100×, in water)**	**g *L*** **^−^** **^1^**	D-Calcium pantothenate	100
L-Proline	136.6 ± 15.3	Thiamine monophosphate	100
		Pyridoxine hydrochloride	100
**Stock solution D (100×, in 1** **M NaOH)**	**mg *L*** **^−^** **^1^**	Riboflavin 5′-phosphate Na	10
L-Histidine	2638±158		
L-Isoleucine	5089±566	**Stock solution I (100×, in 1** **M NaOH)**	**mg *L*** **^−^** **^1^**
L-Asparagine	14,701±1649	Folic acid	100
L-Methionine	703±77		
L-Phenylalanine	3910±435	**Inorganic Salts**	
		**Stock solution J (100×, in water)**	**g *L*** **^−^** **^1^**
**Stock solution E (100×, in 1** **M HCl)**	**mg *L*** **^−^** **^1^**	MgSO_4_	9.8
L-Cystine	2165±155	KH_2_PO_4_	6
L-Leucine	8105±915	Na_2_HPO_4_	19
L-Tyrosine	558±38		
L-Aspartic acid	4851±539	**Stock solution K (25×, in water)**	**g *L*** **^−^** **^1^**
L-Glutamic acid	8859±981	NaCl	200
L-Cysteine	12,000	KCl	10
L-Tryptophan	2000	MgCl_2_	2.3
		CaCl_2_	3.5
**Saccharides**			
**Stock solution F (100×, in water)**	**mg *L*** **^−^** **^1^**	**pH indicator**	
Xylose	468±84	**Stock solution L (100×, in 1** **M NaOH)**	**g *L*** **^−^** **^1^**
Fucose	746±17	Phenol red	1

This optimal SGM is prepared by diluting and mixing various stock solutions and individual nutritional ingredients as follows:

(1) To prepare the 12 kinds of stock solutions (A-L), respectively.
(2) To prepare the ovary extract and eye stalk extract of shrimps, respectively.
(3) To dilute and mix the various stock solutions and individual ingredients one by one.
(4) To isolate and culture the shrimp hemocytes using the optimal SGM.

### Preparation of the stock solutions A-L

For amino acids, as shown in Table 2, there are 5 kinds of stock solutions (A, B, C, D and E) with a concentration of 100× needed to prepare, based on the solubility of amino acids (water-, acid- and alkali-soluble) and their contents in the medium. Of them, stock solution A consists of 12 kinds of water-soluble amino acids of glycine, L‑serine, l-valine, l-lysine, l-alanine, β-alanine, ornithine, l-arginine, l-threonine, hydroxyproline, 4-hydroxybutanoic acid (γ-ABA) and l-glutamine. Stock solution B and C are water-soluble taurine and l-proline, respectively. Stock solution D includes 5 kinds of alkali-soluble amino acids of l-histidine, l-isoleucine, l-asparagine, l-methionine and l-phenylalanine, which are pre-dissolved in 1 M NaOH and then diluted in water. Stock solution E includes 7 kinds of acid-soluble amino acids of l-cystine, l-leucine, l-tyrosine, l-aspartic acid, l-glutamic acid, l-cysteine and l-tryptophan, which are pre-dissolved in 1 M HCl and then diluted in water.

For saccharides, there are 2 kinds of stock solutions F and G with a concentration of 100× needed to prepare. Stock solution F consists of 11 kinds of water-soluble saccharides (xylose, fucose, ribose, sucrose, lactose, fructose, arabinose, trehalose, gentiobiose, *D*+ Galactose and sodium pyruvate). Stock solution G contains only glucose which is water-soluble in water.

For vitamins, there are 2 kinds of stock solutions (H and I) with a concentration of 100× needed to prepare. Stock solution H is made up of 7 kinds of water-soluble vitamins including i-inositol, niacinamide, choline chloride, d-calcium pantothenate, thiamine monophosphate, pyridoxine hydrochloride and riboflavin 5′-phosphate Na. Stock solution I contains only folic acid which is alkali-soluble in 1 M NaOH.

For inorganic salts, there are also 2 kinds of stock solutions (J and K) needed to prepare. Stock solution J is a mixture of 3 kinds of water-soluble inorganic salts of MgSO_4_, KH_2_PO_4_, and Na_2_HPO_4_ with a concentration of 100×. Stock solution K is a mixture of 4 kinds of water-soluble inorganic salts of NaCl, KCl, MgCl_2_ and CaCl_2_ with a concentration of 25×. Instead of a stock solution, the pH regulator salt of NaHCO_3_ is added in powder separately just before used.

The stock solution L which contains phenol red only is prepared as a pH indicator to a concentration of 100×.

All the above-mentioned stock solutions are stored at −80 ℃ until used.

### Preparation of shrimp ovary and eye stalk extracts

**Fig. 1:**
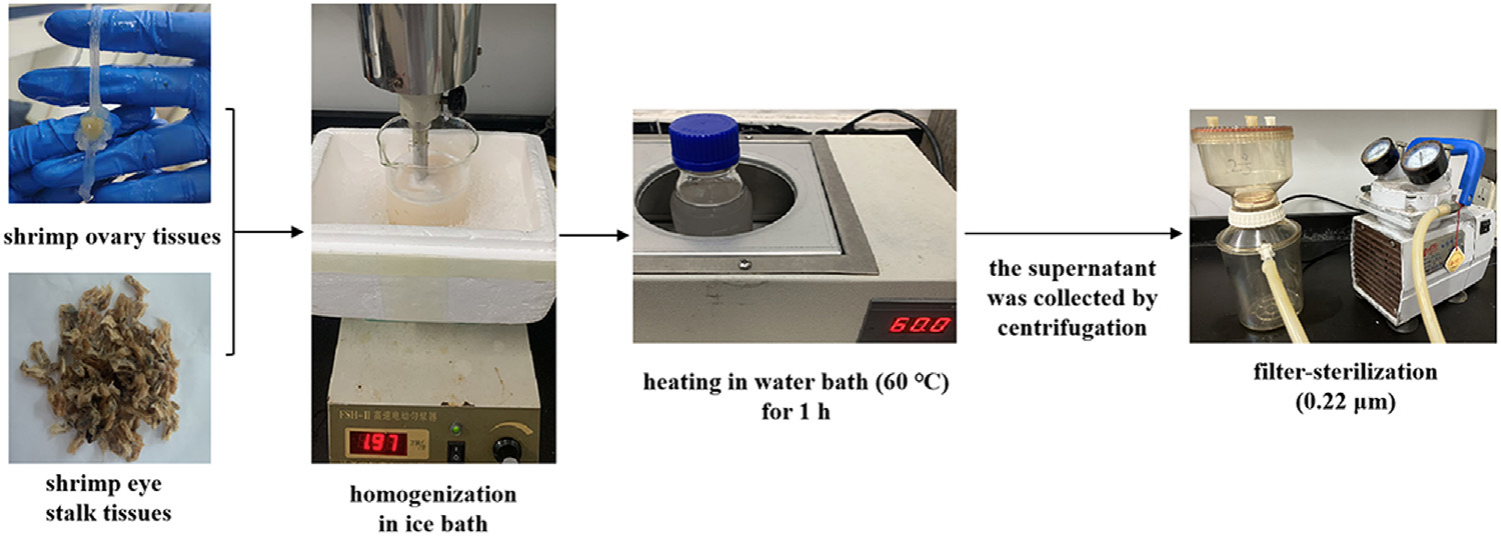
Diagram of the preparation process of shrimp tissue extracts.

As shown in Fig. 1, the shrimp ovary or eye stalk tissues are aseptically dissected and then homogenized in an ice bath after suspended in sterile water at a ratio of 20 mL per gram tissues, respectively, and then incubated in a 60 ℃ water bath for 1 h with intermittent oscillation. The homogenates are frozen and thawed once, and then centrifuged at 10,000 × *g* for 2 h at 4 °C. After that, the supernatant is collected, filter-sterilized (0.22 µm) by a vacuum filtration system (Corning, USA) and stored at −80 ℃ until used [2].

### Preparation of the optimal SGM

**Fig. 2:**
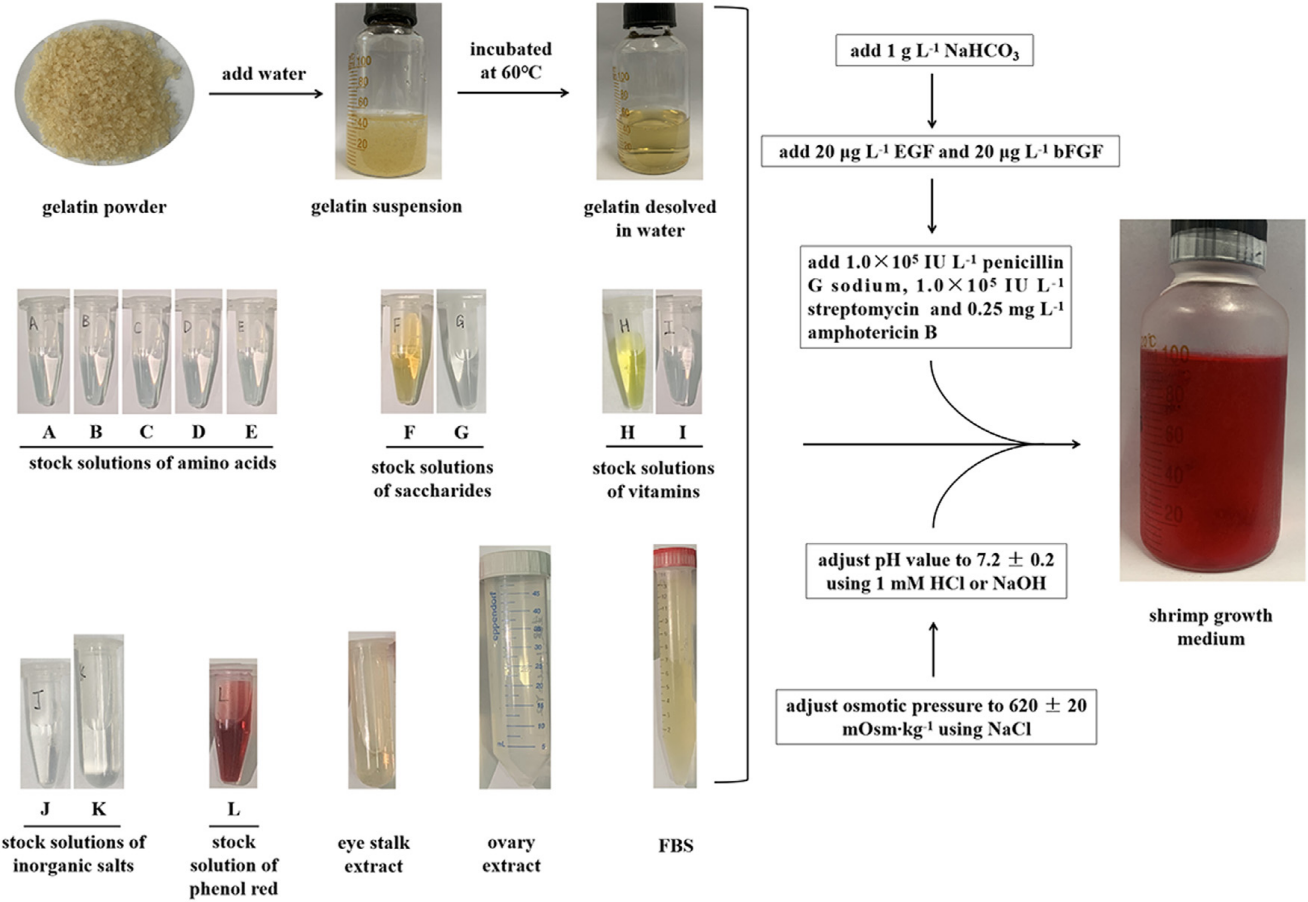
Diagram of the preparation process of the optimal shrimp cell growth medium.

As shown in Fig. 2, the gelatin is a water-insoluble powder at room temperature, but it can dissolve and form an aqueous solution when heated to 60 °C, and then, the aqueous solution of gelatin will gel once it cools [3]. Thus, the aqueous solution of gelatin is the first component to be prepared in case of the possible inactivation or degradation of other nutrients by heat.

**Fig. 3:**
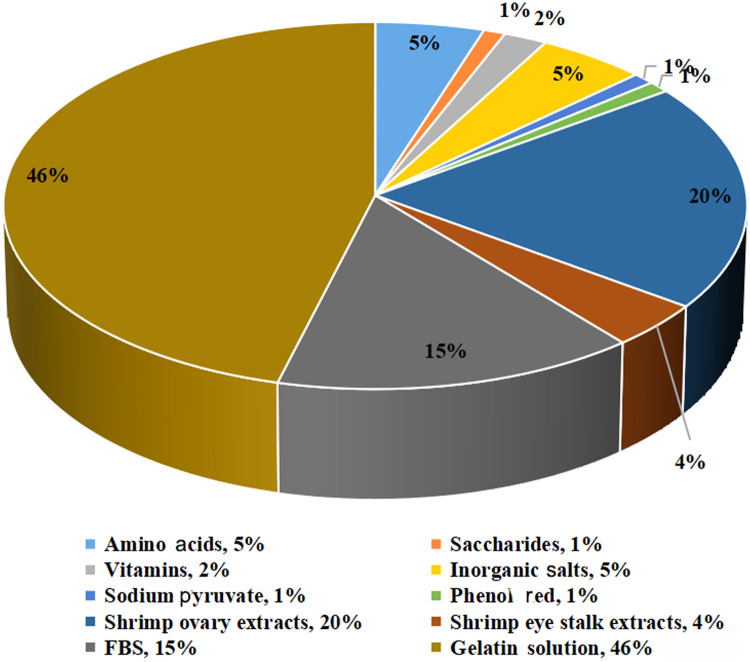
The volume percentages of each of the stock solutions and other supplements in the optimal shrimp cell growth medium.

To prepare a 100 mL medium, forty-six mL of gelatin water solution containing 8.6 g gelatin in powder is first prepared and then cools to 37℃. After that, the 12 kinds of stock solutions (A-L) as listed in Table 2 are diluted and added into the gelatin water solution successively as required and mixed well immediately, followed by the addition of 100 mg NaHCO_3_. After well mixing, the pH value of the medium was adjusted to 7.2 ± 0.2 by 1 M HCl and 1 M NaOH, and the osmotic pressure was adjusted to 620 ± 20 mOsm·kg^−1^ by NaCl, and the volume was made up to 100 mL using sterile water. Next, the medium is filter-sterilized (0.22 µm), and then growth factors (EGF and bFGF), antibiotic mixture (penicillin, streptomycin and amphotericin B) are added into the medium as required just before use. The freshly prepared SGM is stored at 4 ℃ and should be used up within one month. The volume percentage of the stock solutions (A-L) and other supplements are summarized in Fig. 3 and you can use it as a reference in the preparation of SGM.

### Isolation and culture of shrimp hemocytes using the optimal SGM

**Fig. 4:**
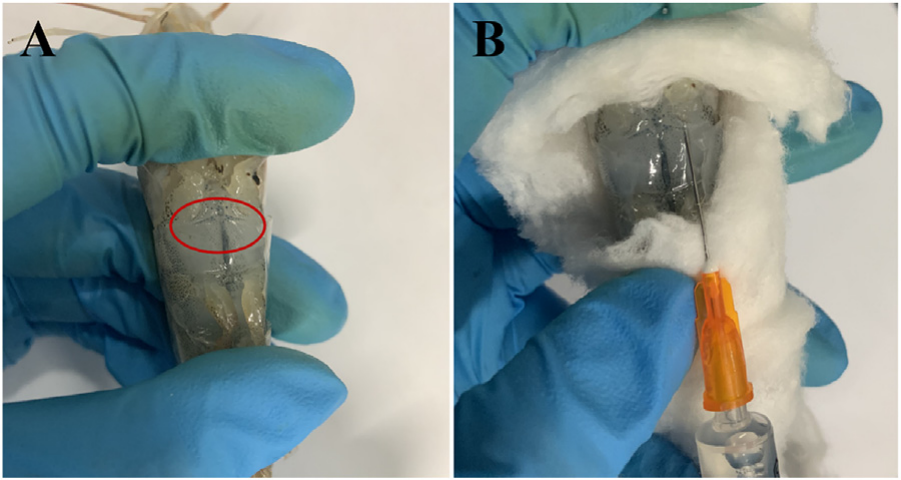
Diagram of the drawing of shrimp hemolymph using a 2.5-mL syringe. Panel A shows the sampling site at the ventral sinus between the pereiopods and pleopods of shrimps as indicated in red circle. Panel B shows the process of drawing out of shrimp hemolymph.

As shown in Fig. 4, after the shrimp is anesthetized by immersing in 75% ethanol for about 10 s, the surface of the sampling area is successively disinfected with 75% alcohol and iodophor solutions [4]. After drying the surface of the sampling area with sterile paper, shrimp hemocytes are aseptically drawn out with a 2.5-mL syringe preloaded with 0.5 mL anticoagulant solution as listed in Table 3. Next, the shrimp hemocytes are pelleted by centrifugation at 1000 × *g* for 10 min and resuspended in 2× PBS (Table 3) containing 30× antibiotic mixtures and treated for 10 min. Then after centrifugation, the cell pellet is pooled and washed once with 2× PBS by centrifugation. Finally, the cell pellet is resuspended with the optimal SGM and seeded into culture plates and incubated at 28℃ with 3% CO_2_, and the medium was half-replaced every 2 or 3 days. Noteworthy, before use, the SGM which is stored at 4 °C refrigerator should be warmed in a water bath to 37℃ to melt it from gelatinous to liquid. It will take ∼15 min for 100 mL SGM to completely melt.

**Table 3:** The formulas of anticoagulant solution (left) and 2× PBS (right).

Anticoagulant Solution		2×PBS	
Components	Concentration (mg *L* ^−^ ^1^)	Components	Concentration (mg *L* ^−^ ^1^)
NaCl	15,000	NaCl	8000
KCl	200	KCl	200
KH_2_PO_4_	200	KH_2_PO_4_	200
Na_2_HPO_4_·12H_2_O	3000	Na_2_HPO_4_·12H_2_O	3000
Trisodium citrate dihydrate	9800		
EDTA·2Na	2480		
Glutathione (or Cysteine)	800 (or 600)		

*Note:* The solutions listed in the table were adjusted for osmotic pressure of 620 ± 20 mOsm·kg^−1^ and pH value of 7.2 ± 0.2.

### Method validation

(1) *Formation of confluent and long-term MeH cell monolayer*

**Fig. 5:**
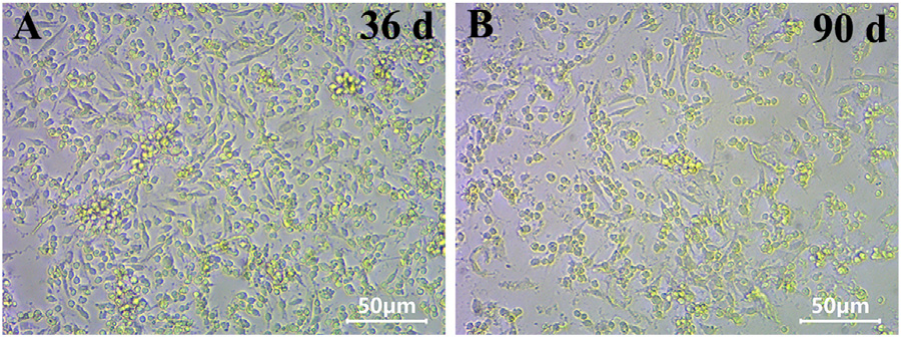
The light micrographs of shrimp hemocytes cultured in the optimal shrimp cell growth medium for 36 (A) and 90 days (B), respectively. Scale bar, 50 µm.

As shown in Fig. 5, using the optimal SGM, confluent MeH cell monolayer can be obtained routinely and healthily maintained for a long time, at least 90 days. The MeH cell type was identified by Wright-Giemsa staining, and data for the cell characterization and cell percentage before and after a long-term culture can be found in the related research article [1]. In brief, SGM can support the long-term survival and growth of three types of shrimp hemocytes, namely granulocytes, semi-granulocytes and prohaemocytes, except for the hyalinocytes which possibly had died or differented into granulocytes or semi-granulocytes in the *in vitro* culture conditions.

Moreover, obvious cytopathic effect (CPE) and replication of shrimp covert mortality nodavirus (CMNV) had been observed in the infected MeH cells cultured in SGM in the test of virus susceptibility, confirming the maintenance of normal cellular functions of shrimp hemocytes in SGM [1].

More information and culture results can be found in the related research article [1].

(2) *Viability analysis of MeH cells by Calcein-AM staining*

As shown in the related research article, the optimal SGM can well support the growth and survival of MeH cells. It was found that by Calcein-AM staining the survival rate of shrimp hemocytes cultured in the optimal SGM for 30 days was 46.24±2.11% [1], much better than previously published results. Calcein-AM (calcein acetoxymethyl ester) can easily penetrate the living cell membrane because of the addition of acetoxymethyl ester (AM) group to Calcein, which enhances its hydrophobicity. Calcein-AM itself can't emit any fluorescence. After entering the cell, it is hydrolyzed by endogenous esterase in the cell to produce a polar molecule of Calcein, which can emit green fluorescence but can't penetrate the cell membrane and be trapped in the cell. In contrast, dead cells can't hydrolyze Calcein-AM into Calcein, thus can't emit fluorescence because of the lack of active esterase in the dead cells [5,6]. In this study, Calcein-AM (Beyotime, Shanghai, China) was used to detect the cellular activity of shrimp hemocytes. Just before staining, the stock solution of Calcein-AM was diluted by serum-free medium into a working concentration of 1 µM. And then the old medium in each well was removed and the cells were washed with 2× PBS for 1–2 times. Then an appropriate volume of Calcein-AM working solution was added, followed by gently shaking in order to make the dye cover all cells, and then incubated for 45 min at 28℃ in darkness. Generally, the added volume of each well of a 96-well plate is 100 µL, that of 24-well plate is 250 µL, that of 12-well plate is 500 µL, and that of 6-well plate is 1 mL, respectively. After incubation, the cells were washed with 2× PBS once, re-fed with fresh medium and observed under an inverted fluorescence microscope.

(3) *Proliferation potential analysis by EdU staining*

As shown in the related research article, the use of the optimal SGM has greatly improved the proliferation potential of shrimp hemocytes. It was found that the proliferation percentages of shrimp hemocytes cultured in the optimal SGM were 30.61±1.92% on the 10th day, but only 0.43±0.14% on the 30th day by EdU staining [1].

EdU (5-ethynyl-2′-deoxyuridine) is a thymidine analogue which can be incorporated into the newly synthesized DNA. The acetylene group of EdU molecule can covalently react with fluorescence-labeled small molecular azide probe (such as Azide Alexa Fluor 488) through the catalysis of univalent copper ions and form a stable triazole ring, which is very rapid and thus called “click reaction”. Through the “click reaction”, the newly synthesized DNA will be labeled with the corresponding fluorescence probe, so that the proliferating cells can be detected using the appropriate fluorescence detection equipment [7]. The BeyoClick™ EdU Cell Proliferation Kit with Alexa Fluor 488 (Beyotime, China) was chosen for use in this study. The reagents required in the experiment but not provided in the kit were shown in Table 4 and the experimental steps were as follows in detail (take a 6-well plate as an example).

a. The medium containing 1× EdU (10 µM), that is, EdU working solution, was added to a well which is seeded with MeH cells and incubated at 28℃ for 24 h.
b. After EdU labeling, the culture medium was removed, and 1 mL of fixative was added and fixed for 15 min at room temperature.
c. The fixative was removed and the cells were washed 3 times with 1 mL of washing solution per well, 3–5 min each time.
d. The washing solution was removed, and each well was incubated with 1 mL permeabilization reagent for 10–15 min at room temperature.
e. The permeabilization reagent was removed, and the cells were washed 1–2 times with 1 mL washing solution for 3–5 min each time.
f. The click reaction solution needed to be ready-made. Added and mixed 430 µL click reaction buffer, 20 µL CuSO_4_, 1 µL azide 488 and 50 µL click additive solution in turn, and used the click reaction solution within 15 min.
g. The washing solution from the step e was removed and then 0.5 mL of click reaction solution was added to each well, and the culture plate was gently shaken to ensure that the reaction mixture could cover the cells evenly.
h. After incubation at room temperature for 30 min in the darkness, the click reaction solution was removed and the cells were washed with the washing solution for 3 times, 3–5 min for each time.
i. In order to detect the cell proliferation percentage, Hoechst 33,342 was used for nuclear staining. 1× Hoechst 33,342 solution was prepared of by diluting the stock solution of Hoechst 33,342 (1000×) with PBS at the ratio of 1: 1000.
j. After the step h, after the washing solution was removed, 1 mL of 1×Hoechst 33,342 solution was added to each well and incubated at room temperature in darkness for 15 min.
k. The 1×Hoechst 33,342 solution was removed and the cells were washed with the washing solution for 3 times, 3–5 min each time. Then fluorescence detection can be carried out.

**Table 4:** The reagents required in EdU assay (not provided in the kit).

Reagents	Preparation
Fixative	3.7% Formaldehyde in PBS
Permeabilization Reagent	0.3% Triton® X-100 in PBS
Washing Solution	3% Bovine serum albumin (BSA) in PBS, pH 7.4
2×PBS	Seen in Table 3

From the results of Calcein-AM staining and EdU staining in the related research article [1], it is found that there is a drastic variation existed between cellular activity and proliferative activity of the MeH cells. It has been reported that shrimp cells usually stop dividing and enter a “quiescence state” once isolated and cultured *in vitro* within for 24 h, that is, the cells are alive but not mitotic [8,9]. In the present study, the SGM has been found to be able to partially solve the problem of mitosis-arrest of shrimp hemocytes, but it is still unable to support the hemocytes to actively proliferate for too long time, which is the next important scientific question to be solved in the future.

There is no doubt that SGM can successfully supported the long-term *in vitro* culture of hemocytes of *Metapenaeus ensis*, but this medium has not been tested in other shrimp species. In consideration of the similar nutrient requirement, we believe that, this medium should be suitable for the hemocyte culture of other seawater penaeid shrimps and even freshwater penaeid shrimps after a minor modification of its osmolarity. For example, based on some literature reports, it was found that the content of proline, taurine and glucose in the sera of some seawater shrimps like *Penaeus vannamei* [10] and *Penaeus stylirostris* [11] was similar to that in the serum of *Metapenaeus ensis*. Of course, the applications of SGM medium in other shrimp species with more distant relationship like lobster and crayfish or other crustacean should be cautious and needs more experimental verification.

## CRediT authorship contribution statement

**Yaqi Zhao:** Methodology, Validation, Data curation, Writing – original draft. **Huarong Guo:** Supervision, Writing – review & editing.

## Declaration of Competing Interest

The authors declare that they have no known competing financial interests or personal relationships that could have appeared to influence the work reported in this paper.

## Data Availability

Data will be made available on request.
